# Melatonin Alleviates Retinal Ischemia–Reperfusion Injury by Inhibiting p53–Mediated Ferroptosis

**DOI:** 10.3390/antiox12061173

**Published:** 2023-05-29

**Authors:** Fan Zhang, Bingying Lin, Siyu Huang, Pengsen Wu, Min Zhou, Jing Zhao, Xiangqing Hei, Yu Ke, Yiting Zhang, Danping Huang

**Affiliations:** State Key Laboratory of Ophthalmology, Zhongshan Ophthalmic Center, Sun Yat-sen University, Guangzhou 510060, China

**Keywords:** retinal ischemia, melatonin, ferroptosis, p53, neuroprotection

## Abstract

Retinal ischemia–reperfusion (RIR) injury caused by high intraocular pressure (IOP) is an important risk factor contributing to retinal ganglion cell (RGC) death, eventually causing blindness. A key progressive pathological process in the development of RIR is the death of RGCs. However, the detailed mechanisms underlying RGC death caused by RIR have not yet been clearly elucidated, and effective treatments are lacking. Ferroptosis is a recently defined form of programmed cell death that is closely related to organ injury. Melatonin (MT) is a promising neuroprotective agent, but its effects on RIR injury remain unclear. In this study, murine models of acute ocular hypertension and oxygen and glucose deprivation/reoxygenation (OGD/R) model were adopted to simulate retinal ischemia. MT alleviated retinal damage and RGC death in RIR mice, significantly attenuating RIR–induced ferroptosis. Furthermore, MT reduced the expression of p53, a master regulator of ferroptosis pathways, and the upregulation of p53 promoted ferroptosis and largely abolished the neuroprotective effects of MT. Mechanistically, the overexpression (OE) of p53 suppressed the expression of the solute carrier family 7 member 11 (Slc7a11), which was accompanied by increased 12–lipoxygenase (Alox12) expression, triggering retinal ferroptosis. Moreover, MT–ameliorated apoptosis, neuroinflammation and microglial activation were observed. In summary, MT conferred neuroprotection against RIR injury by inhibiting p53–mediated ferroptosis. These findings indicate that MT is a retina–specific ferroptosis inhibitor and a promising therapeutic agent for retinal neuroprotection.

## 1. Introduction

Retinal ischemia–reperfusion (RIR) injury is implicated in several ocular diseases, such as glaucoma, diabetic retinopathy (DR) and retinal vascular occlusions [1,2,3]. Retinal ganglion cell (RGC) death is a common event in these diseases that causes irreversible damage to visual function [1,2]. Although much effort has been devoted to exploring effective therapies aiming to attenuate RGC death, glaucomatous damage to RGCs can progress despite intraocular pressure (IOP) reduction. Therefore, alternative neuroprotective strategies are urgently needed to improve the survival and function of RGCs. However, the precise pathogenesis of RGC death has not been thoroughly elucidated.

Ferroptosis is an emerging type of cell death driven by the lethal accumulation of iron–dependent lipid peroxidation and plays an important role in ischemic injury in important human organs, including the heart [4], brain [5], kidney [6], and lung [7]. The inhibition of ferroptosis has been shown to be an efficient approach for treating multiple organ damage [6,7]. Oxidative stress induced by excessive iron deposition plays an important role in the progression of ferroptosis. Notably, substantial evidence suggests that oxidative stress is a prominent contributor to the pathogenesis of RIR injury [8]. Thus, ferroptosis caused by oxidative stress and excessive iron deposition may contribute to the promotion and progression of RIR injury. Hence, the therapeutic targeting of ferroptosis may be a promising strategy for RGC protection in RIR injury.

Acetyl–5–methoxytryptamine (melatonin, MT) is a neurohormone that has been widely investigated as a promising neuroprotective agent due to its metal chelation, anti–lipid peroxidation, anti–inflammatory, and antioxidative properties [9,10]. A recent study demonstrated that MT rescued the brain function of mice after traumatic brain injury [11]. Another recent publication reported that MT attenuated acute kidney injury by inhibiting ferroptosis through its effects on the nuclear factor erythroid 2–related factor 2 (Nrf2)/solute carrier family 7 member 11 (Slc7a11) axis [12]. RGCs are vulnerable to oxidative stress and lipid peroxidation due to their low levels of antioxidant enzymes and high levels of polyunsaturated fatty acids (PUFAs) [13,14]. Therefore, treatments that attenuate oxidative stress and reduce lipid peroxidation may provide protection against the progression of RGC death. However, the question of whether and how MT affects the iron–dependent form of regulated RGC death is still unclear.

Increasing evidence suggests that p53 plays a crucial role in modulating ferroptosis through its multipotent effects on lipid reactive oxygen species (ROS), iron, and amino acid levels [15,16]. In some cancer cells, the expression of Slc7a11 is severely suppressed by p53 activation, which results in intracellular cysteine depletion and sensitizes the cells to ferroptotic cell death [17]. Moreover, MT has previously been demonstrated to regulate the expression of p53 and genes involved in p53 signaling [18]. As expected, our preliminary mechanistic investigations revealed that MT markedly prevented the specific binding of p53 to the Slc7a11 promoter. Thus, it is reasonable to speculate that MT may restrain RIR injury by inhibiting p53–mediated ferroptosis.

This work demonstrates, for the first time, the antiferroptotic effect of MT on RIR injury, as well as the detailed regulatory pathways through which MT regulates ferroptosis, suggesting its role as a potential novel therapy to rescue RGCs from death induced by RIR.

## 2. Materials and Methods

### 2.1. Human Eye Tissue Subject Study

With the approval of the Ethics Committee of the ZOC, Sun Yat–sen University, and in accordance with the Declaration of Helsinki, human retinas were collected from deceased healthy donors and end–stage glaucoma patients (who required for removal of intraocular contents). Healthy donor eyes were obtained anonymously from the human tissue biobank of Zhongshan Ophthalmic Center, Sun Yat–sen University. Written signed informed consent was obtained from the participants or their respective proxies. The retinas were fixed in 4% paraformaldehyde (PFA) for later staining with hematoxylin and eosin (H&E) staining and used for immunofluorescence assays. Table S1 lists the antibodies used in this study. Confocal fluorescence images were captured using an LSM880 microscope (Carl Zeiss, Hallbergmoos, Germany). For the human retinas, western blotting was performed using standard procedures.

### 2.2. Experimental Design and Drug Administration

Six– to eight–week–old C57BL/6J male mice were purchased from Guangdong Medical Laboratory Animal Center (Guangzhou, China) and were fed on a regular basal diet and maintained in accordance with the Association for Research in Vision and Ophthalmology Statement on Animal Welfare. The protocol was approved by the Ethics Committee of ZOC and Sun Yat–sen University. The in vivo experiment was conducted in four parts. In each part of the experiment, the mice were grouped using the random number table method. In the first part of the experiment, ferrostatin–1 (Fer–1), a specific ferroptosis inhibitor, was used as the treatment, and the mice were divided into the following groups: the sham group, sham+Fer–1 (2 µg/kg) group, RIR+Vehicle group, and RIR+Fer–1 (2 µg/kg) group. In the second part, the following groups were evaluated: the sham group, sham+MT (100 µg/kg) group, RIR+Vehicle group, RIR+Low–dose MT (50 µg/kg) group, and RIR+High–dose MT (100 µg/kg) group. The intraocular delivery of MT (MedChemExpress, Monmouth Junction, NJ, USA) or Fer–1 (MedChemExpress) was administered 1 h before anterior chamber perfusion using a 5 µL 33G syringe needle (Hamilton, Reno, NV, USA). MT or Fer–1 was first dissolved in dimethyl sulfoxide (DMSO) and then in PBS. The final DMSO concentration was 1%. PBS containing the same concentration of DMSO served as the vehicle control. In the third part of the experiment, the antagonists (Sigma Aldrich, St. Louis, MO, USA) were dissolved in 2.5% *v*/*v* ethanol/PBS, as described in a previous study [11]. Luzindole (luz, a nonspecific MT1/MT2 antagonist) and 4–phenyl–2–propionamidotetralin (4–P–PDOT, a MT2–selective antagonist) were administered 1 h before retinal ischemia via intraperitoneal (i.p.) injection, and the mice were divided into the following groups: the RIR+Vehicle group, RIR+MT group, RIR+MT+luzindole (10 mg/kg) group, and RIR+MT+4–P–PDOT (10 mg/kg) group. In the fourth part of the experiment, mice subjected to RIR were used to study the role of p53 in the following groups: the adeno–associated virus 2 (AAV2)–NC+Vehicle group, AAV2–NC+MT group, AAV2–p53+Vehicle group, and AAV2–p53+MT group. AAV2–p53 and AAV2–GFP were designed and packaged by Vigene (Jinan, China). One microliter (10^12^ vg/mL) of AAV2 was injected into the vitreous body four weeks prior to ischemia based on a prior report [19].

### 2.3. Mouse RIR Injury Model

The mice were deeply anesthetized with pentobarbital sodium (35 mg/kg) via i.p. injection. Then, tetracaine hydrochloride (0.5%) was used for corneal surface anesthesia, and tropicamide (1%) was applied to dilate the pupils. A 32–gauge needle for insulin injection was inserted into the anterior chamber of the eyes and connected to a 0.9% saline infusion system, and the IOP was elevated to 110 mmHg for 70 min by increasing the height of the saline bag to 150 cm. The IOP of the mice was measured using an iCare rebound tonometer (TonoLab, Icare, Helsinki, Finland) (Figure S1). During the modeling process, hypromellose eye drops (Zhongshan Ophthalmic Center, Guangzhou, China) was applied to keep the cornea moist. The eyes of the sham mice were cannulated and maintained at a normal IOP. Tobramycin ointment (Alcon, Fort Worth, TX, USA) was placed over the eyes to minimize the risk of infection.

### 2.4. Histological Analysis of Retinal Tissue and Retinal Ganglion Cell (RGC) Survival Analysis

Seven days after reperfusion, histological analysis and retinal whole–mounts were obtained to investigate RIR–induced morphological changes and RGC loss. The mice were overdosed with pentobarbital and transcardially perfused with isotonic saline followed by 4% PFA. Next, the eye tissue was removed and embedded in 4% PFA and then sliced into serial paraffin sections across the optic disc. Then, the eyes were enucleated, fixed, and stained with H&E. Retinal thickness measurements were performed on sections within 1 mm of the optic nerve center. The number of cells within a 200 µm length of the ganglion cell layer (GCL) was counted in 10 fields (40× magnification) of 2 sections for each eye. The surviving RGCs were labeled using retinal flat mount immunofluorescence and quantitatively represented. Briefly, the eyes were removed and further fixed in 4% PFA for approximately 2 h. Next, the retinas were dissected from the eyeballs, blocked and permeabilized with 0.5% Triton X–100 and 10% normal donkey serum (Solarbio, Beijing, China) for 10 min, and then incubated overnight at 4 °C with antibodies against neuron–specific beta3–tubulin (Tuj–1) and RNA–binding protein with multiple splicing (RBPMS). The next day, the retinas were washed with PBS and incubated with the corresponding Alexa Fluor 488–, 594–, or 647–conjugated secondary antibody for 2 h at room temperature in the dark. The retinas were mounted with antifade mounting medium (Solarbio) after washing with PBS, and images were taken using a Zeiss LSM880 confocal microscope (Carl Zeiss). Subsequently, the total average number of RGCs was calculated in all eight fields, including four quadrants and eight areas. The number of RGCs in each image was counted using ImageJ (NIH, Bethesda, MD, USA). 

### 2.5. Spectral Domain Optical Coherence Tomography (OCT) Imaging of Live–Mouse Retinas

Seven days after modeling, OCT was performed with a protocol consisting of a circular scan centered on the optic nerve head using a Spectralis HRA+OCT+Multicolor (Heidelberg Engineering, Heidelberg, Germany). The mice were anesthetized, and their pupils were dilated as described above. The thicknesses of the ganglion cell complex (GCC) and the whole retina (the RNFL to RPE) were measured and then analyzed independently by two skilled observers, who were blinded to the subsequent groups, using ImageJ.

### 2.6. Primary RGC Culture and Treatment

Primary mouse RGCs were obtained from the retinas of mouse pups (from postnatal day 1 [P]1 to P3) using a two–step immunopanning method according to a previously published protocol [20]. Specifically, retinal samples were separated from the mice to prepare single–cell suspensions. First, the isolated samples were incubated with lectin from Bandeiraea simplicifolia (Vector Laboratories, Burlington, ON, Canada) to remove non–neuronal cells. The nonadherent cells were transferred to flasks coated with Thy1.2 monoclonal antibody (Bio–Rad, Oxford, UK). The purified RGCs were incubated with RGC–conditioned medium containing supplements. The experimental cells were pretreated with MT or Fer–1 for 6 h and then subjected to OGD/R. The cells were then cultivated in glucose–free DMEM and transferred to a modular incubator chamber (Billups–Rothenberg, San Diego, CA, USA) infused with a gas mixture consisting of 95% N_2_ and 5% CO_2_, incubated at 37 °C for 3 h, and then incubated in high–glucose DMEM and returned to the incubator under normoxic conditions for 12 h. The drug concentrations were kept the same before and during the OGD/R process.

### 2.7. Cell Viability Assay

The effects of the drug on cell viability were monitored using a Cell Counting Kit–8 (CCK–8, APExBIO Technology, Houston, TX, USA), as described previously [21]. The primary RGCs were seeded into 96–well plates and cultured with 100 μL RGC–conditioned medium. Different concentrations of MT (1, 5, 25, 50 μM) were added to the wells, and the cells were incubated for 24 h in a 5% CO_2_ atmosphere. Ten microliters of CCK–8 solution was added, and the cells were incubated for another 3 h at 37 °C. Finally, the absorbance was evaluated at 450 nm using a microplate reader (Synergy H1 Hybrid Reader, BioTek, Potton, UK).

### 2.8. Cell Death Assay

The cells were collected and stained with calcein–AM/propidium iodide (Beyotime Biotechnology, Shanghai, China) after treatments. Briefly, the isolated RGCs were incubated with PI (1×) and calcein–AM (1×) for 30 min at 37 °C. The fluorescence was visualized using a fluorescence microscope (Nikon, Tokyo, Japan). The percentage of living cells was quantified by counting the PI–positive (dead) cells and calcein–AM–positive (viable) cells. The investigators who measured the percentage of living cells were blinded to the treatment groups.

### 2.9. Real–Time Polymerase Chain Reaction (qRT–PCR)

Total RNA was extracted from the tissues using an RNA purification kit (YISHAN Biotechnology, Shanghai, China) and reverse–transcribed into cDNA using Evo M–MLVRT Premix for qRT–PCR (Accurate Biology, Changsha, China). qRT–PCR was performed with the SYBR Green Premix Pro Taq HS qPCR Kit (Accurate Biology) using a LightCycler ^®^ System (Roche, Pleasanton, CA, USA). The samples were subjected to the following program: 45 cycles of 95 °C for 15 s, 65 °C for 10 s, and 72 °C for 10 s. The relative gene expression was calculated using the 2^−ΔΔCT^ method. All primer sequences are summarized in Table S2.

### 2.10. Western Blot Analysis

Retinal homogenates were prepared in ice–cold lysis buffer supplemented with PMSF, protease inhibitor cocktail, and phosphatase inhibitor cocktail (all from KeyGen BioTECH, Nanjing, China) with a tissue homogenizer and cleared via centrifugation (12,000× g for 10 min at 4 °C). The retinal protein concentration was determined using bicinchoninic acid (BCA) kit (KeyGen BioTECH), and equal amounts of total protein were electrophoresed on 10% polyacrylamide gel and subsequently transferred to polyvinylidene fluoride (PVDF) membranes (Millipore, Billerica, MA, USA) following a standard protocol. After blocking for 10 min with QuickBlock™ Blocking Buffer (Genscript, Nanjing, China), the membranes were incubated with primary antibodies for 4 °C overnight followed by incubation with HRP–conjugated secondary antibodies for 2 h at room temperature. After washing three times with 0.1% Tween–20/TBS, the immunoreactive proteins were visualized with an enhanced chemiluminescence kit (Millipore) and a chemiluminescence imager (Tanon Science & Technology, Shanghai, China). For stripping, the membranes were incubated in a mild stripping buffer (Epizyme Biomedical Technology, Shanghai, China) for 10 min when necessary. The recorded data and densitometric analysis were quantified using ImageJ. The relative expression of a specific protein was calculated with respect to β–Actin.

### 2.11. Measurement of Iron, Malondialdehyde (MDA), and Glutathione (GSH) Levels in Retinal Tissue Samples

The levels of iron concentration, MDA, and GSH in the retinal tissues were measured using an iron assay kit (Abcam, Cambridge, UK), MDA assay kit (NanJing JianCheng Bioengineering Institute, Nanjing, China), and GSH assay kit (Solarbio), respectively, according to the respective manufacturers’ protocols.

### 2.12. Immunohistochemical, Immunofluorescence Staining, and TUNEL Assay

After routine fixation, primary RGCs or retinal tissue slices were subjected to timed incubation, and immunostaining was performed according to standard procedures using antibodies. A TUNEL assay was carried out using the Dead End TUNEL system (Elabscience, Shanghai, China) according to the manufacturer’s instructions. The images in the figures were acquired using confocal microscopy (Carl Zeiss).

### 2.13. Analysis of the Mitochondrial Membrane Potential (MMP) via JC–1 Staining

MMP changes in the RGCs were assessed with JC–1 (Beyotime) according to the manufacturer’s instructions, and the changes were assessed and recorded using a confocal microscope (Carl Zeiss) and flow cytometry (BD, Franklin Lakes, NJ, USA). A decrease or increase in the red/green fluorescence intensity ratio represents a decrease or increase in the MMP (Δψm).

### 2.14. Transmission Electron Microscopy (TEM)

For TEM, the retinal samples (1 mm × 1 mm × 1 mm) were quickly removed and fixed via immersion in cold 2.5% glutaraldehyde. These samples were postfixed, embedded, cut, and further sliced into 80 nm thick sections. A Hitachi H–7500 transmission electron microscope (Hitachi, Tokyo, Japan) was utilized to analyze the morphology of the retinas, including their ultrastructure.

### 2.15. Measurement of Lipid Peroxidation In Vitro

To measure OGD/R–induced lipid peroxidation in the RGCs, the primary RGCs were exposed to OGD/R for indicated times and treated with drugs. After washing with PBS, 5 μM C11–BODIPY 581/591 (Invitrogen, Carlsbad, CA, USA) was added to each well, and the cells were incubated at 37 °C for 30 min in the dark. The nuclei were labeled with Hoechst 33258 (Solarbio). Lipid peroxidation was assessed using a confocal microscope (Carl Zeiss) and flow cytometry (BD). Green fluorescence indicated oxidation reactions.

### 2.16. ChIP–PCR Assay

We used a Pierce Agarose ChIP kit (Thermo Fisher Scientific, Rockford, IL, USA), mainly following previously described protocols [22]. An anti–p53 antibody or IgG antibody was used for immunoprecipitation. Then, DNA was extracted and analyzed via PCR gel electrophoresis and qRT–PCR was performed.

### 2.17. Coimmunoprecipitation (Co–IP) Assay

A co–IP assay was performed as described previously [23]. Briefly, retinal tissue lysates were incubated with the antibodies at 4 °C overnight, and then, protein A/G magnetic beads (Millipore) were added to the lysates. The eluted immunocomplexes were subjected to SDS–PAGE and immunoblotted with anti–Slc7a11 and anti–12–lipoxygenase (Alox12) antibodies.

### 2.18. Statistical Analysis

The data are presented as the mean ± SD. The results were statistically analyzed using SPSS 22.0 software. The two–tailed Student’s t–test and one–way ANOVA followed by Tukey’s post hoc test were used to compare two different groups and more than two groups, respectively. A *p* value < 0.05 indicated statistical significance.

## 3. Results

### 3.1. Significant RGC Loss and the Expression of Ferroptosis Markers in Enucleated Eyes from Glaucoma Patients

**Figure 1:**
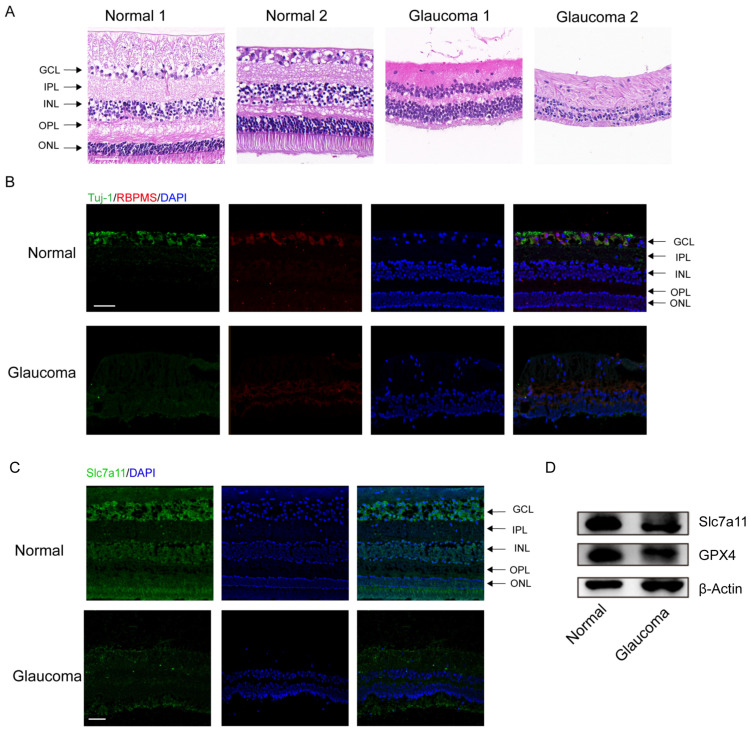
Significant RGC loss in enucleated eyes from glaucoma patients. (**A**) Analysis of morphological changes in human retinas via H&E staining (scale bar = 20 μm). (**B**) Images of immunofluorescence labeling of the RGC–specific markers Tuj–1 (green) and RBPMS (red) from human retinal tissues (scale bar = 50 μm). (**C**) Immunofluorescence images of expressed Slc7a11 protein (green) and 4′,6–diamidino–2–phenylindole (DAPI) staining (blue) in healthy donor and glaucomatous retinas (scale bar = 50 μm). (**D**) The levels of the ferroptosis–related proteins GPX4 and Slc7a11 were detected via western blotting.

The clinical characteristics of the glaucoma patients are summarized in Table S3. The results of H&E staining demonstrated significant RGC loss and retinal disorganization in the donors with end–stage glaucoma (Figure 1A). In addition, the number of RBPMS– and Tuj–1–labeled RGCs was obviously reduced among the donors with end-stage glaucoma compared to the healthy control subjects (Figure 1B). Through immunofluorescence staining, we measured the protein levels of Slc7a11, a negative regulator of ferroptosis in retinas. The Slc7a11 immunofluorescence staining intensity was decreased in the retinas of patients with glaucoma compared with those of those nonglaucoma donors (normal subjects) (Figure 1C). Western blot analysis showed that the expression of Slc7a11 and GPX4 in the retinas of the glaucoma patients was downregulated compared with the healthy control subjects (Figure 1D). 

### 3.2. Fer–1 Pretreatment Attenuated RIR–Induced Retinal Damage

**Figure 2:**
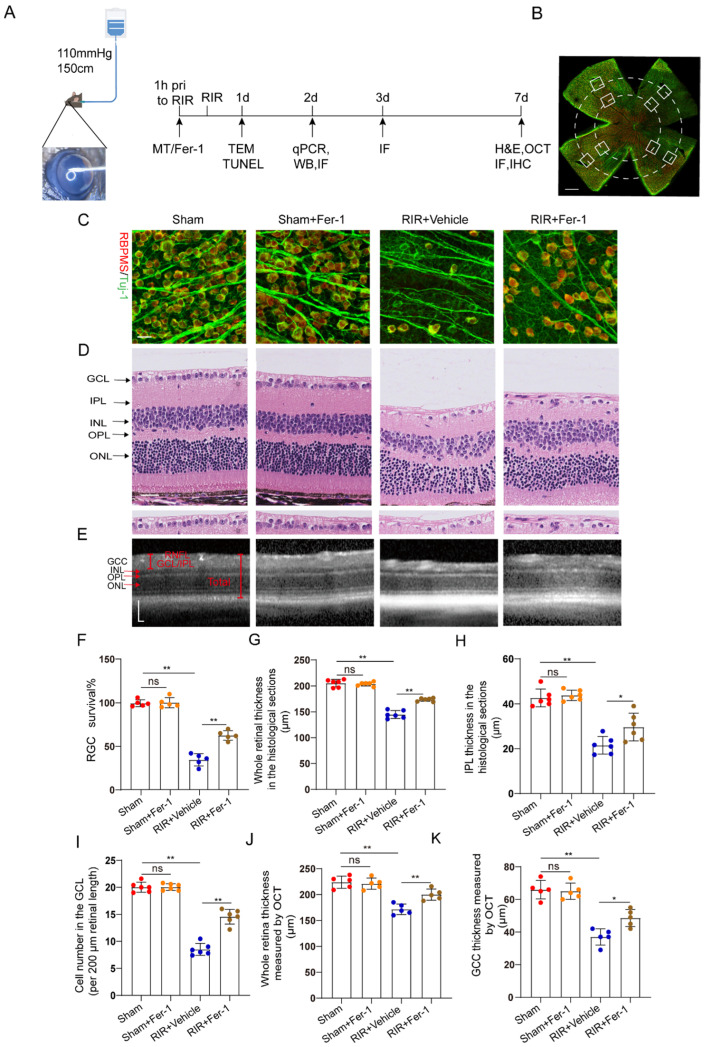
Fer–1 attenuated retinal damage by inhibiting ferroptosis in mice subjected to RIR. (**A**) A mouse model of acute ocular hypertension and the timeline of the experimental design. (**B**) Eight images of the peripheral and middle areas of the retinal flat mounts were captured from each mouse (scale bar = 500 μm). (**C**) Images of mouse retinas after immunofluorescence staining for the RGC markers Tuj–1 (green) and RBPMS (red) (scale bar = 25 μm). (**D**) Typical images of H&E–stained mouse retinas (scale bar = 25 μm). (**E**) Representative OCT images of live mice showing retinal morphology after RIR with or without Fer–1 (scale bar = 100 μm). (**F**) The percentage of viable Tuj–1+ and RBPMS+ RGCs in retinal flat mounts normalized to that observed in the retinas of the sham mice (n = 5). (**G**–**I**) Analysis of the whole retinal thickness, IPL thickness, and cells in the GCL via H&E staining (n = 6). (**J**,**K**) The bar charts represent quantitative measurements of the GCC thickness and whole retina thickness in live mice, as determined via OCT (n = 5). The colored dots on the graphs represent data points for different individuals from the following groups: the Sham (red), Sham+Fer–1 (orange), RIR+Vehicle (blue), and RIR+Fer–1 (brown) groups. All the results are presented as the mean ± SD and were analyzed using one–way ANOVA followed by Tukey’s post hoc test; ns = not significant, * *p* < 0.05, ** *p* < 0.01.

Increasing evidence suggests that ferroptosis drives IR–induced neuronal death [24,25]. Fer–1, a potent inhibitor of ferroptosis, was used to confirm whether Fer–1 can alleviate high–IOP–induced retinal damage by inhibiting ferroptosis (Figure 2A). Compared with the vehicle, Fer–1 administration led to the retention of a considerable number of RGCs, as shown by the RGC markers captured from the retinal flat mounts (Figure 2B) after immunofluorescence staining (Figure 2C,F). In addition, images of the H&E–stained mouse retinas showed that unlike the vehicle, Fer–1 protected the whole retina from becoming thinner after RIR. The thickness of the inner plexiform layer (IPL, which contains the dendritic arbors of the RGCs) was decreased after RIR, but this change was ameliorated through Fer–1 supplementation (Figure 2D,G–I). Compared with those from the sham mice, retinal sections from the RIR mice showed a decrease in the number of cells in the GCL (containing the cell bodies of the RGCs), but the number of cells in the GCL was increased by the Fer–1 pretreatment. In addition to the histological analysis of the isolated retinas, the measurement of the GCC thickness in the live mice retinas was also assessed quantitatively, as the GCC thickness is often used as a morphological indicator of dynamic RGC damage. Consistent with the H&E staining results, MT treatment significantly prevented the decreases in the whole retinal thickness and GCC thickness, as determined via OCT (Figure 2E,J,K). Collectively, these results clearly demonstrate that ferroptosis is activated during the process of RIR and can be effectively blocked by Fer–1.

### 3.3. MT Pretreatment Attenuated Retinal Damage after RIR

**Figure 3:**
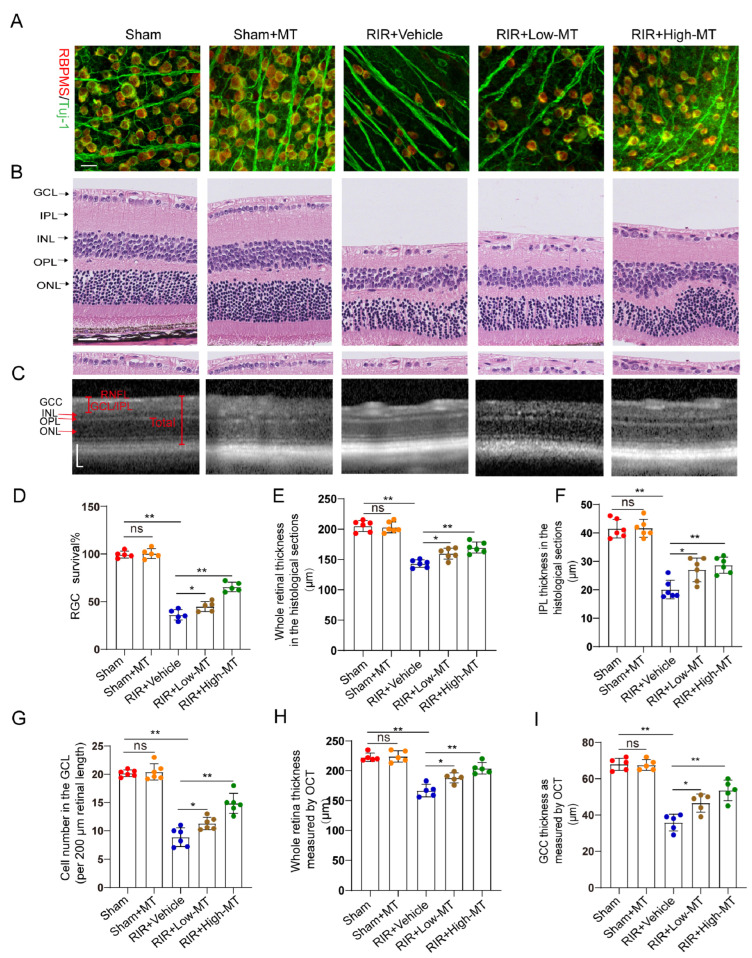
MT attenuated retinal damage and RGC loss after RIR. (**A**) Immunofluorescence images of RGCs stained for Tuj–1 (green) and RBPMS (red); representative images are shown (scale bar = 25 μm). (**B**) Representative images of H&E–stained retinal sections from the different groups (scale bar = 25 μm). (**C**) Retinal morphology and thickness in MT–pretreated and unpretreated live mice were assessed noninvasively with OCT (scale bar = 100 μm). (**D**) Quantitative analyses of RBPMS– and Tuj1–positive RGCs in the mice retinas of the different groups (n = 5). (**E**–**G**) Quantitative analyses of the whole retinal thickness, IPL thickness, and number of cells in the GCL in mice of the different groups based on H&E staining of the retinal sections (n = 6). (**H**,**I**) Quantitative analyses of the whole retinal thickness and GCC thickness in the live mice of the different group as determined via OCT (n = 5). The colored dots on the graphs represent data points for different individuals from the following groups: the sham (red), sham+MT (orange), RIR+Vehicle (blue), RIR+Low–MT (brown), and RIR+High–MT (green) groups. All the results are presented as the mean ± SD and were analyzed using one–way ANOVA followed by Tukey’s post hoc test; ns = not significant, * *p* < 0.05, and ** *p* < 0.01.

Evidence has shown that MT has a clear protective effect against cell death [26]. In this study, the effects of MT on the survival of RGCs were assessed by counting the number of specific stain (Tuj–1 and RBPMS)–positive cells in retinal flat mounts. It is evident from the analysis that the number of Tuj–1–and RBPMS–positive RGCs in the retina was significantly reduced after RIR. However, an increase in RGC survival was apparent following pretreatment with MT, and survival varied with the MT concentration in a dose–dependent manner (Figure 3A,D). Next, the thickness of the whole retina and IPL and the cell density in the GCL were assessed through H&E staining to evaluate the effect of MT on retinal morphology changes (Figure 3B). A comparison of the retinal thickness and cell density in the GCL between the sham and sham+MT groups revealed no change. The retinas exposed to RIR displayed a significant marked reduction in the number of retinal cells in the GCL and retinal thickness, especially the retinal IPL thickness. The damage observed in the retina was significantly mitigated by MT. While both the low–dose and high–dose groups showed improving trends toward the alleviation of these changes, the alterations were significantly ameliorated in the high–dose group (Figure 3G–I). RIR caused a progressive and significant thinning of the thickness of whole retina and the GCC, and MT pretreatment ameliorated these changes, as measured with OCT (Figure 3C,H,I), further verifying that MT reduced the degree of retinal tissue loss after RIR. These results demonstrated that MT markedly increased retinal structure preservation and RGC survival after RIR.

### 3.4. Melatonin Attenuated RIR–Induced Ferroptosis

**Figure 4:**
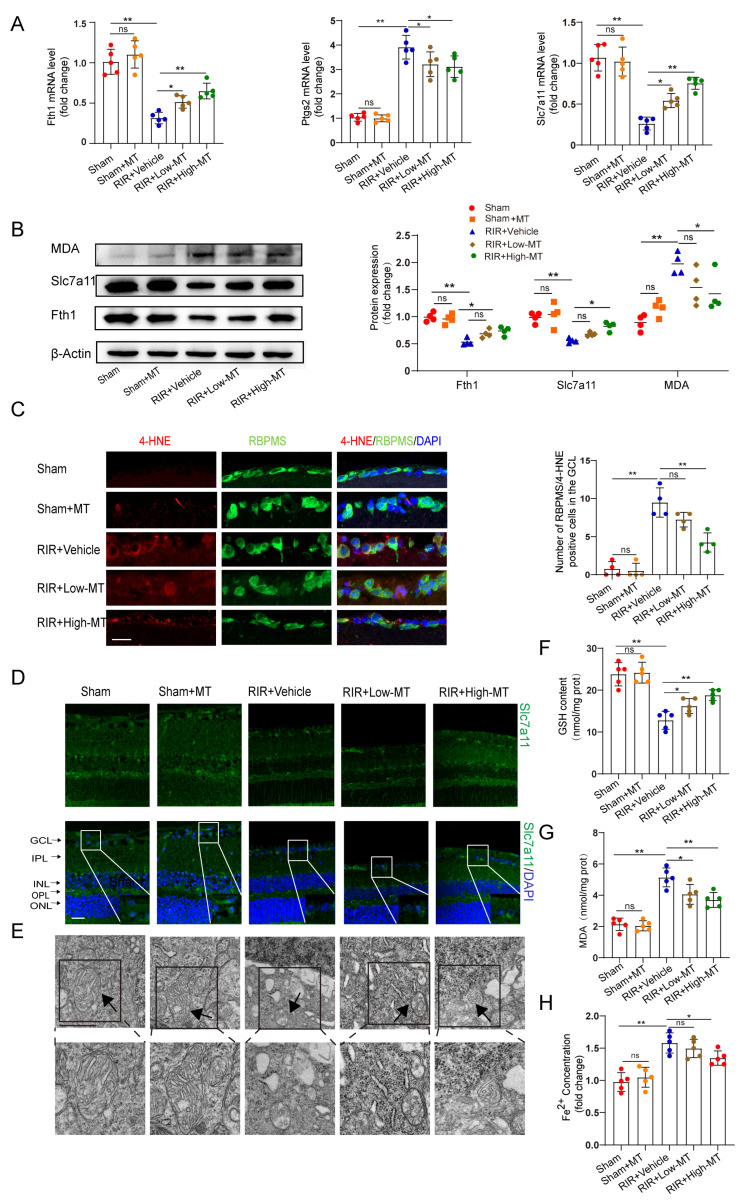
MT ameliorated RIR–induced ferroptosis. (**A**) qRT–PCR analysis of the mRNA expression of Fth1, Slc7a11, and Ptgs2 (n = 5). (**B**) Representative Western blotting analysis for the quantification of the protein levels of the ferroptosis markers Fth1, Slc7a11, and MDA. β–Actin was used as the loading control (n = 4). (**C**) The number of lipid–peroxidation–marker (4–HNE)–positive RGCs in the GCL of the mouse retinal sections was assessed via immunofluorescence staining (n = 4) (scale bar = 25 µm). (**D**) Images of immunofluorescence staining of Slc7a11 in the mouse retina (scale bar = 25 µm). (**E**) Ultrastructural analysis of mouse retinas from each group (scale bar = 1 µm); the arrows indicate mitochondria (n = 5). (**F**,**G**) The levels of GSH and MDA, were measured in the retinas after RIR (n = 5). (**H**) Fe^2+^ levels in the retinas of mice in the different groups were assessed (n = 5). The colored dots on the graphs represent data points for different individuals from the following groups: the sham (red), sham+MT (orange), RIR+Vehicle (blue), RIR+Low–MT (green), and RIR+High–MT (brown) groups. All the results are presented as the mean ± SD and were analyzed via one–way ANOVA followed by Tukey’s post hoc test; ns = not significant, * *p* < 0.05, ** *p* < 0.01.

Recent evidence suggests that the MT–mediated inhibition of ferroptotic cell death can ameliorate tissue damage [11,12]. Next, we measured the effects of two concentrations of MT on the mRNA and protein levels of some putative ferroptosis–related biomarkers. The transcript levels of the ferroptosis marker genes ferritin heavy chain (Fth1) and Slc7a11 were decreased, while a significant upregulation of prostaglandin–endoperoxide synthase 2 (Ptgs2) expression was observed in the RIR mice. The mRNA expression of Ptgs2 was markedly inhibited, while the Fth1 and Slc7a11 levels were increased in the retinal tissues of the MT–pretreated mice compared to those of mice in the RIR group (Figure 4A). The differences in the Fth1 and Slc7a11 protein levels were roughly consistent with the changes in transcript levels between the high–dose MT group and the RIR group. However, low–dose MT failed to rescue the protein levels of Fth1 and Slc7a11 (Figure 4B). Both MDA and 4–hydroxynonenal (4–HNE) are very common end products of lipid peroxidation and have been shown to be directly involved in inducing ferroptosis [27]. The MDA level in the retinal tissues was measured with western blotting and an MDA assay kit, and the results showed that the MDA level was reduced by a high dose of MT after RIR (Figure 4B,G). In addition, compared with that in the sham mice, the number of 4–HNE–positive RGCs was increased after RIR, while pretreatment with a high dose of MT significantly decreased the number of 4–HNE–positive RGCs, as shown by 4–HNE staining. However, the number of 4–HNE–positive RGCs did not differ significantly between the low–dose MT and RIR groups (Figure 4C). Consistent with what we previously found in the human retinas, the immunofluorescence assay results showed a decreased Slc7a11 level in the RIR mice, and this decrease was reversed through MT supplementation (Figure 4D). Then, the GSH content was evaluated. As expected, a decrease in the GSH levels was observed after modeling, and this decrease was blocked by MT (Figure 4F). The alteration of mitochondrial morphology is a typical feature of ferroptosis [28]. TEM revealed that the mitochondria of the normal RGCs exhibited an intact structure, including an abundant mitochondrial matrix and many cristae, but the mitochondria were small, the cristae were reduced, and the membrane density was increased in the IR–injured retinas, suggesting mitochondrial damage and dysfunction. MT supplementation reversed these ferroptosis–related changes, including decreases in the mitochondrial size and mitochondrial ridges (Figure 4E). These outcomes suggest that MT may help to rescue mitochondrial function and morphology. In addition, excessive iron, an abundant and powerful redox–active oxidant, catalyzes the Fenton reaction during intracellular ROS formation and thus causes cell death [29,30]. Hence, iron chelation may prevent ferroptosis. As expected, high–dose MT administration significantly reduced the intracellular level of Fe^2+^ that had accumulated in the IR–injured retinas. Despite the fact that the change was also observed in the RIR mice to which we administered a low dose of MT, the intracellular level of Fe^2+^ was not significantly different between the low–dose MT group and the RIR group (Figure 4H). Altogether, these results showed that pretreatment with MT markedly rescued RIR–induced lipid peroxidation and oxidative stress and attenuated the damage to the mitochondrial structure, especially when administered at a high dose. Therefore, a high dose of MT was selected for the subsequent in vivo experiments.

### 3.5. MT Promoted Primary Cultured RGC Survival, Restored MMP, and Inhibited Lipid Peroxidation following OGD/R

**Figure 5:**
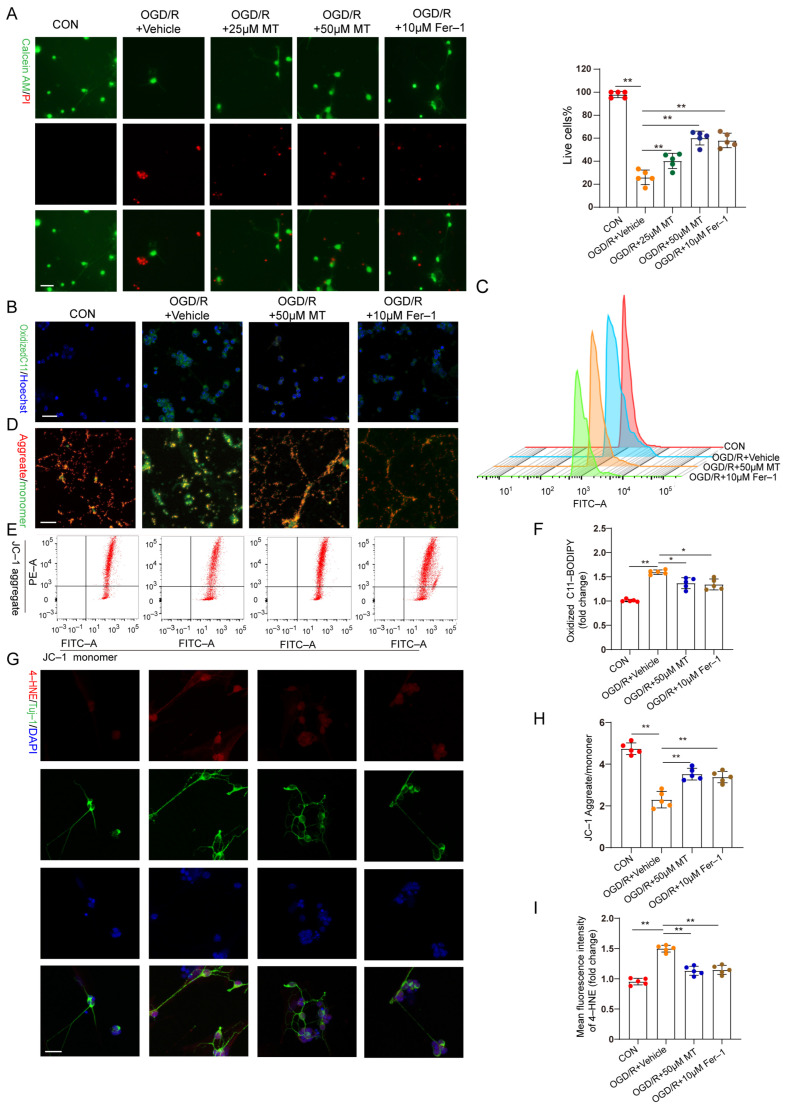
MT significantly and specifically promoted primary RGC survival after OGD/R exposure. (**A**) Representative immunofluorescence images of dead (red) and live (green) cells obtained through the double staining of primary RGCs with calcein–AM and PI in primary RGCs after OGD/R (n = 5) (scale bar = 25 μm). (**B**,**C**) Measurement of lipid ROS levels identified with BODIPY 581/591 C11 using immunofluorescence staining and flow cytometry (scale bar = 25 μm). (**D**,**E**) The MMP in primary RGCs was detected via JC–1 staining and analyzed via flow cytometry (scale bar = 25 μm). (**F**) Statistical analysis of lipid ROS levels (n = 5). (**G**) Images of primary RGCs after immunofluorescence staining for 4–HNE (red) and Tuj–1 (green) in each group (n = 5) (scale bar = 10 μm). (**H**) Statistical analysis of the MMP levels (n = 5). (**I**) Statistical analysis of the fluorescence intensity of 4–HNE. The colored dots on the graphs represent data points for different types of interventions from the following groups: CON (red), OGD/R+Vehicle (orange), OGD/R+25 μL MT (green), OGD/R+50 μL MT (blue), and OGD/R+10 μL Fer–1 (brown). All the results are presented as the mean ± SD and were analyzed using one–way ANOVA followed by Tukey’s post hoc test; * *p* < 0.05, ** *p* < 0.01.

In vitro, OGD/R–induced RGC injury was used to study the protective effect of MT on RGCs. RGCs were isolated, and the characteristic markers Brn3a and RBPMS were analyzed (Figure S2A). A CCK–8 assay revealed that 1 to 50 µM MT exerted no obvious toxic effects on the primary RGCs (Figure S2B). Cell survival decreased profoundly, and the cell death rate increased significantly after OGD/R but was substantially ameliorated after treatment with different doses of MT and Fer–1 (Figure 5A). Next, we observed the impacts of MT and Fer–1 on changes in the MMP and lipid ROS levels after OGD/R. The lipid ROS levels were further analyzed and visualized using C11–BODIPY staining and flow cytometry. Intense green fluorescence, representing oxidized C11–BODIPY, was observed. We confirmed that OGD/R increased the lipid ROS levels (Figure 5B,C,F). MMP collapse has been recognized as a sign of cellular damage that is accompanied by ferroptosis and intrinsic apoptosis [31]. JC–1 staining and flow cytometry showed severe impairment of the MMP in the OGD/R group (Figure 5D,E,H). However, supplementation with MT and Fer–1 significantly reduced the ROS production induced by OGD/R and partially restored the MMP. Moreover, significantly more intense immunofluorescence staining of 4–HNE was observed in the OGD/R group compared to the MT– and Fer–1–treated groups (Figure 5G,I). These in vitro results confirmed that MT directly increased RGC survival, restored the MMP, and protected cells from lipid peroxidation after OGD/R.

### 3.6. MT Alleviated RGC Apoptosis Induced by RIR Injury

**Figure 6:**
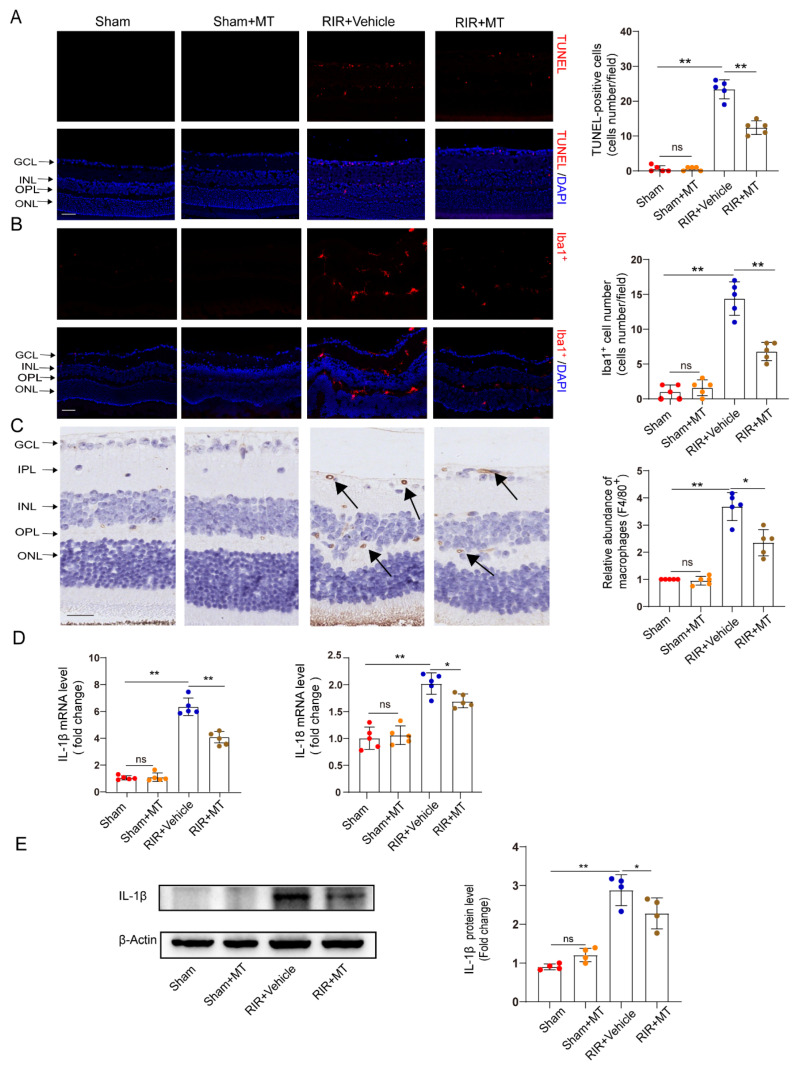
MT ameliorated neuronal apoptosis, reactive gliosis, and the inflammatory response after RIR. (**A**) Representative immunofluorescence images of TUNEL–stained frozen retinal sections taken from mice (n = 5) (scale bar = 50 μm). (**B**) Representative images of immunofluorescence staining of Iba1–expressing microglia (red) and DAPI (blue) staining of mouse retinal slices and quantitative analyses of Iba1^+^ cells in the retina (n = 5) (scale bar = 50 μm). (**C**) Representative images of F4/80^+^ immunohistochemical staining of the mouse retina. The arrows indicate F4/80^+^ cells (n = 5) (scale bar = 25 μm). (**D**) The mRNA levels of IL–1β and IL–18 were calculated via qRT–PCR analysis (n = 5). (**E**) The protein expression of IL–1β in the retina was determined via Western blotting (n = 4). The colored dots on the graphs represent data points for different individuals from the following groups: the sham (red), sham+MT (orange), RIR+Vehicle (blue), and RIR+MT (brown) groups. All the results are presented as the mean ± SD and were analyzed using one–way ANOVA followed by Tukey’s post hoc test. ns = not significant, * *p* < 0.05, ** *p* < 0.01.

RGC apoptosis is involved in RIR injury [32]. TUNEL staining revealed a significant increase in the number of TUNEL–positive cells in the GCL and INL of the RIR group compared with the sham group, and it generally decreased to a lower level in the mice subjected to RIR after pretreatment with MT (Figure 6A). Our results demonstrated that MT significantly prevented RGC apoptosis induced by RIR.

### 3.7. MT Ameliorated Reactive Gliosis and the Inflammatory Response after RIR Injury

Neuroinflammation and microglial activation induced by RIR leads to RGC death [33,34]. To investigate whether MT pretreatment alleviates microglia–mediated neuronal damage, we specifically examined microglial activation in the retina by measuring the number and distribution of Iba1^+^ cells. Iba1^+^ cells were found in almost all the retinal layers after RIR. A marked increase in the number of Iba1^+^ cells was observed in the RIR mice compared to the sham group, while a significant decrease in the number of Iba1^+^ cells in the MT–pretreated mice was observed (Figure 6B). To quantify the number of macrophages in the retina, macrophages were stained with a known macrophage marker, F4/80^+^ [35]. The F4/80^+^–stained cells accumulated in the IR retinas, but the accumulation of F4/80^+^ cells was reversed by the MT pretreatment (Figure 6C). The expression of the proinflammatory cytokines IL–1β and IL–18 was lower in the MT pretreatment group than in the RIR group, as determined via qRT–PCR (Figure 6D). Additionally, higher IL–1β protein levels were found in the mice subjected to RIR, but MT pretreatment significantly attenuated the protein level of IL–1β (Figure 6E). Based on the aforementioned results, MT treatment effectively attenuated microglia/macrophages activation and reduced severe retinal inflammation.

### 3.8. The Protective Effect of MT against RIR Injury through MT Receptors

**Figure 7:**
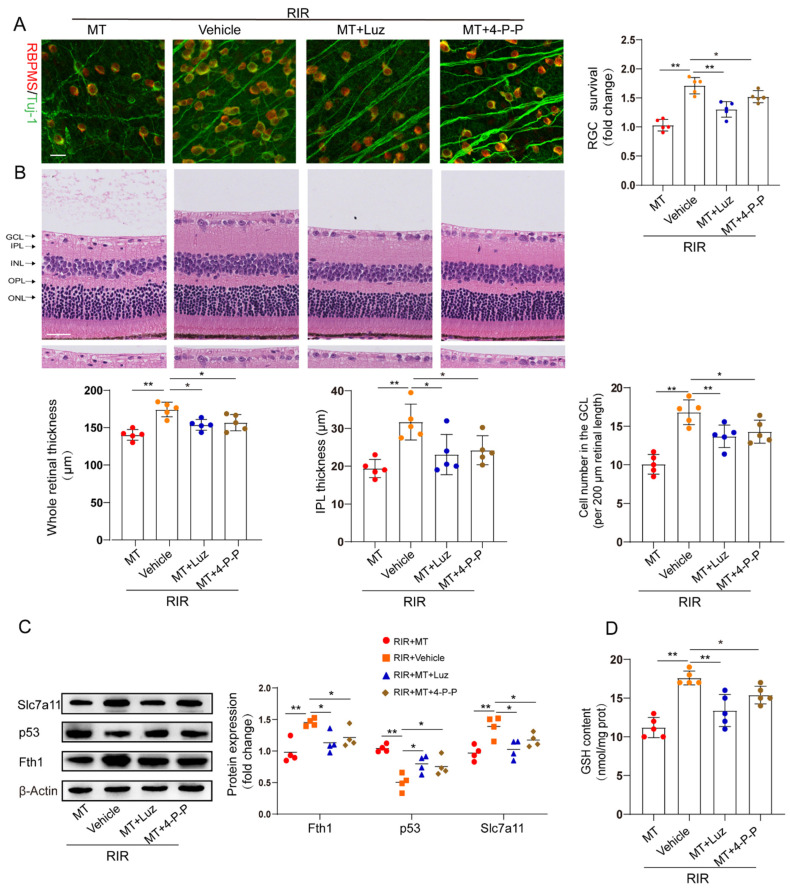
MT receptors were involved in MT–conferred protection against RIR injury. (**A**) Analysis of RGC survival via staining for Tuj–1 (green) and RBPMS (red) in retinal flat mounts from mice (n = 5) (scale bar = 25 µm). (**B**) Representative images of H&E–stained mouse retinal sections (n = 5) (scale bar = 25 µm). (**C**) Western blotting showing Fth1, Slc7a11, and p53 expression in injured retinas from different groups following RIR (n= 4). (**D**) GSH contents in retinal tissues. The colored dots on the graphs represent data points for different individuals from the following groups: the RIR+Vehicle (red), RIR+MT (orange), RIR+MT+Luz (blue), and RIR+MT+4–P–PODT (brown) groups. All the results are presented as the mean ± SD and were analyzed via one–way ANOVA followed by Tukey’s post hoc test. * *p* < 0.05, ** *p* < 0.01. Luz, luzindole; 4–P–P, 4–P–PDOT.

To determine whether MT receptors are involved in RIR–induced ferroptosis, we pretreated mice with luzindole and 4–P–PDOT before the onset of retinal ischemia. The restorative effects of MT on the RGC number and retinal thickness were partly abolished by both luzindole and 4–P–PDOT (Figure 7A,B). The increase in p53 expression and the decrease in the expression of the ferroptosis markers Fth1 and Slc7a11 in the RIR mice were ameliorated by MT. However, the inhibitory effect of MT on p53 expression and its ability to increase the Slc7a11, Fth1, and GSH contents were reversed by the MT receptor antagonists’ administration in RIR injury (Figure 7C,D).

### 3.9. MT Pretreatment Attenuated RIR–Induced Ferroptosis by Mediating the p53/Slc7a11/Alox12 Axis

**Figure 8:**
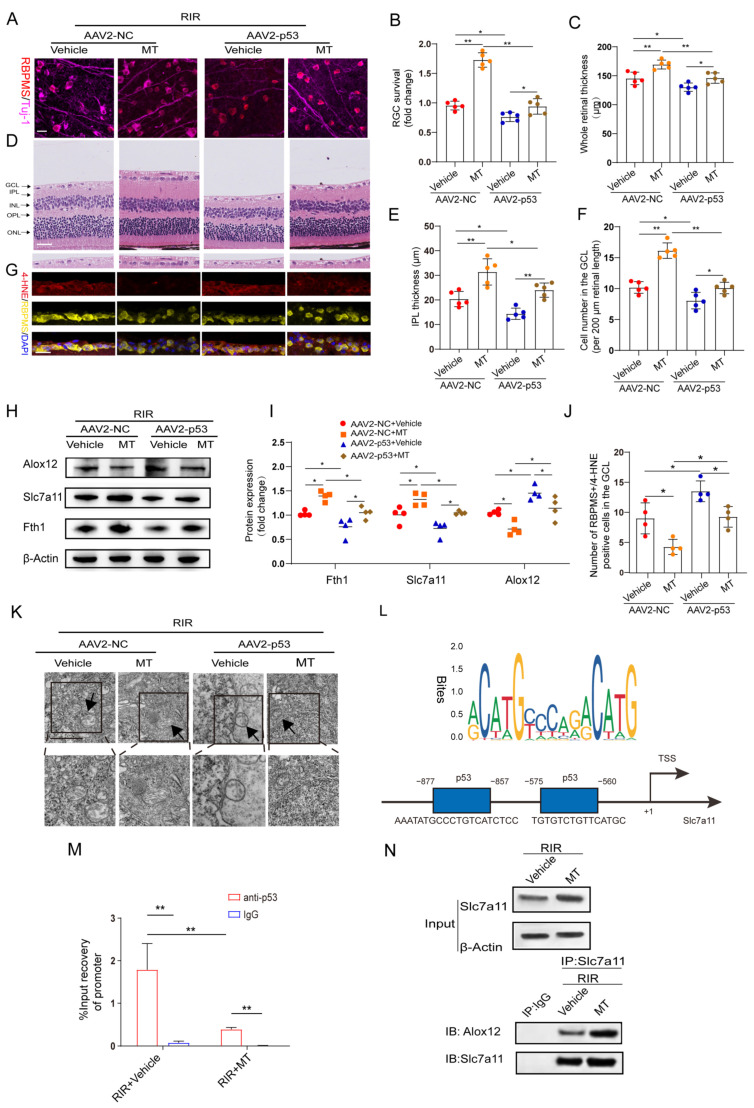
MT treatment attenuates RIR–induced ferroptosis by mediating the p53/Slc7a11/Alox12 axis. (**A**) Images of retinal flat mounts from mice with p53 OE (scale bar = 25 μm). (**B**) H&E staining of retinas from mice treated with or without MT was performed after AAV2–p53 or AAV2–NC injection (scale bar = 25 μm). (**C**) 4–HNE staining was performed, and the number of 4–HNE–positive RGCs in the ganglion cell layer (GCL) in the mouse retinal sections was determined (scale bar = 25 μm). (**D**) Fold changes in RGC survival were quantified 1 week post–operation (n = 5). (**E**–**G**) Quantification of the degree of retinal damage in H&E–stained retinal sections (n = 5). (**H**,**I**) Western blot analysis of the protein levels of ferroptosis markers after p53 overexpression. β–Actin was used as the loading control (n = 4). (**J**) Quantification of 4–HNE–positive RGCs in the GCL (n = 4). (**K**) Ultrastructural analysis of the retinas of mice treated with or without MT after RIR following AAV2–p53 injection. The arrows indicate mitochondria (n = 5) (scale bar = 1 μm). (**L**) The predicted transcription factor (TF) motif of p53 and binding sites on the slc7a11 host gene promoter, which was identified via the JASPAR database. (**M**) ChIP–PCR assays were performed to detect the binding of p53 to the promoter of Slc7a11 in the retinas of mice treated with or without MT following RIR injury. (**N**) Co–IP results showing the endogenous interaction between Slc7a11 and Alox12 in mice subjected to RIR and treated with or without MT. The colored dots on the graphs represent data points for different individuals from the following groups: the AAV2–NC+Vehicle (red), AAV2–NC+MT (orange), AAV2–p53+Vehicle (blue), and AAV2–p53+MT (brown) groups. All the results are presented as the mean ± SD and were analyzed via one–way ANOVA followed by Tukey’s post hoc test. * *p* < 0.05, ** *p* < 0.01.

According to the aforementioned results, MT significantly suppressed the expression of p53, which was previously confirmed as a positive regulator of ferroptosis [15]. Therefore, we speculated that the inhibitory effect of MT on ferroptosis is involved in its repressive effect on p53 expression. To test this hypothesis, p53 was overexpressed in retinas before inducing retinal ischemia. Green fluorescence was observed in the whole retina and was found to accumulate particularly in the RGCs after AAV2−GFP injection, indicating a good transduction efficiency (Figure S3A). In addition, the protein level of p53 was detected 4 weeks after AAV2−p53 injection (Figure S3B). Furthermore, our results revealed that p53 overexpression (OE) exacerbated RIR−induced RGC death and significantly attenuated MT−ameliorated retinal thickness (Figure 8A–F). Next, the levels of the ferroptosis−associated 4−HNE protein were also evaluated after RIR, and fewer 4−HNE−positive RGCs were observed in the GCL of the AAV2−NC group, while p53 OE suppressed the effect of MT in decreasing the number of 4–HNE−positive RGCs (Figure 8G,J). To clarify the regulatory effect of MT on the inhibition of ferroptosis via the p53 pathway, ferroptosis−related protein expression was measured. As expected, the results of western blotting revealed that p53 OE markedly decreased the expressions of Slc7a11 and Fth1 and increased Alox12 expression as compared to the AAV2−NC group. Moreover, the effects of the application of MT on the levels of ferroptosis–related proteins were reversed by p53 OE (Figure 8H,I). The application of MT clearly improved the morphological changes in the mitochondria resulting from RIR, as indicated by a more visible mitochondrial structure with almost fully intact cristae. Conversely, p53 OE inhibited the effect of MT in ameliorating the changes in mitochondrial morphology, as indicated by the disappearance of the mitochondrial cristae and an increased membrane density (Figure 8K). In addition, compared to the AAV2−NC group, a progressive decline in the GSH contents was observed in the p53 OE group, and the MT–mediated increase in the GSH levels was reversed by p53 OE (Figure S3C). When p53 was overexpressed, the MDA levels were promoted, and the decrease in the MDA levels caused by MT was reversed (Figure S3D). All these results suggest that p53 OE inhibits the antiferroptotic effects exerted by MT, contributing to RIR progression. To gain an in–depth understanding of, and further clarify, the specific molecular mechanism of MT–mediated ferroptosis regulation, the downstream targets of p53 were explored. Bioinformatics analysis with Jaspsar (JASPAR: http://jaspar.genereg.net, accessed on 1 December 2022) indicated two binding sites of p53 on the Slc7a11 promoter (Figure 8L). To determine whether p53 and Slc7a11 exert regulatory effects, ChIP–PCR was performed, and the results showed that p53 occupied the promoter of Slc7a11 to directly repress Slc7a11 transcription. Moreover, MT markedly prevented p53 binding to the promoter of Slc7a11. These observations suggest that Slc7a11 is a potential transcriptional target of p53–mediated transcriptional repression and that MT can relieve this transcriptional repression in RIR injury (Figure 8M). Furthermore, previous works have linked the repression of Slc7a11 to the activation of Alox12, which is involved in p53–mediated ferroptosis [36]. Thus, co−IP analysis was performed. The results revealed that Slc7a11 and Alox12 interacted with each other in vivo and that Slc7a11 exhibited a stronger interaction with Alox12 in the MT−pretreated mice than in the RIR mice (Figure 8N). On the basis of these mechanistic studies, MT inhibited p53−mediated ferroptosis by promoting Slc7a11 at the transcriptional level and repressing Alox12 expression via the restoration of Slc7a11 expression. Considering the aforementioned results, p53 OE inhibited the beneficial effect of MT in inhibiting ferroptosis. Accordingly, it can be inferred that MT rescued Slc7a11 expression by downregulating p53, which contributed to an increase in the GSH level and a decrease in the Alox12 level, thus inhibiting ferroptosis.

## 4. Discussion

**Figure 9:**
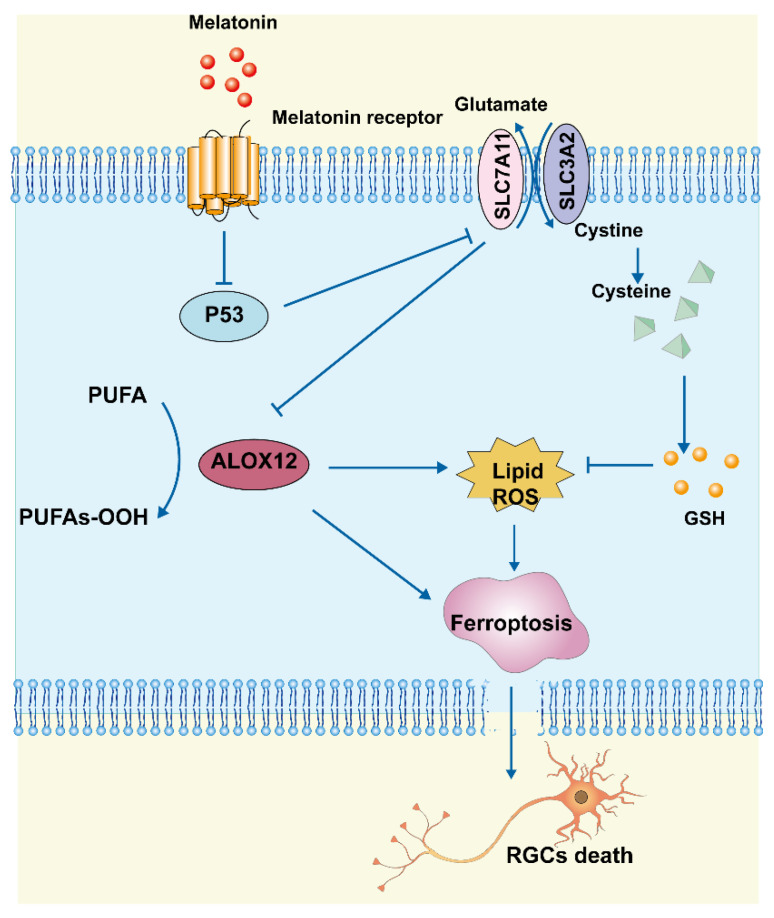
Schematic representation of the neuroprotective mechanisms through which MT inhibits ferroptosis by regulating the p53/Slc7a11/Alox12 axis in RIR injury.

In this study, we found that MT reduced RGC death after RIR by inhibiting ferroptosis, apoptosis, and inflammation. Morphologically, MT administration increased mitochondrial structural components in the mouse retina. Biochemical analyses showed that MT attenuated the decrease in GSH levels, reduced lipid accumulation, and inhibited the level of Fe^2+^. Mechanistic research further revealed that MT rescued Slc7a11 expression by downregulating p53, which contributed to a decrease in Alox12 expression, thereby inhibiting ferroptosis. These findings demonstrate a novel antiferroptotic mechanism of MT, with MT inhibiting ferroptosis via the p53/Slc7a11/Alox12 pathway in RIR injury (Figure 9).

The use of MT to treat neuronal injury is garnering interest, because MT has been reported to be an antioxidant and neuroprotective agent that ameliorates oxidative stress by scavenging ROS and stimulating the production of endogenous antioxidant enzymes [37,38]. Thus, MT shows potential to be used for the treatment of neuronal injury of the inner retina. A wide range of studies have examined the effects of MT on retinal functions and confirmed its protective effects on the retina. Jiang et al. [39] demonstrated that MT protected the retina by inhibiting the production of proinflammatory cytokines and retinal lipid peroxidation in DR. In our study, the first relevant finding suggested that the intravitreal administration of endogenous MT provided significant structural neuroprotection following RIR, which indicates that MT is a valuable therapeutic agent for RGC neuroprotection.

As a novel mode of regulatory cell death, ferroptosis is triggered by the inactivation of cellular GSH–dependent antioxidant defenses, leading to the iron–dependent accumulation of toxic lipid ROS, which influences the pathological processes of various diseases [27,40]. In this study, we found that retinal tissues displayed some interesting hallmarks after RIR, including abnormal iron metabolism, lipid peroxidation, and GSH deficiency. Notably, these hallmarks represent key biochemical phenomena required for ferroptosis. During tissue ischemia/hypoxia, lipid peroxides can enhanced oxidative stress and inflammation [41,42,43]. The study presented here confirmed that MT inhibits RIR–induced lipid peroxidation, which indicates that MT acts as an effective lipid ROS scavenger. Importantly, system Xc–mediated cystine uptake is critical for GSH synthesis and the main mechanism that prevents lipid peroxidation, facilitating the restoration of redox homeostasis and increasing cell survival [44,45]. Our study revealed a decrease in the Slc7a11 protein level in the retina after RIR. Thus, targeting Slc7a11 may be an effective treatment option for protecting RGCs from ferroptosis. Previous studies have shown that the upregulation of ATF4 increases the expression of Slc7a11 and maintains intracellular GSH levels to reduce oxidative stress in HT22 hippocampal cells [46]. Our study showed that MT elevated the GSH levels in RIR mice, thereby improving resistance to oxidative stress. The regulation of ferritinophagy and regulation of iron metabolism are additional potential mechanisms underlying the control of RIR–associated injury [47]. Consistent with prior research, we found that retinal iron was accumulated and Fth1 expression was downregulated following RIR, indicating that iron storage was reduced, and Fe^2+^ was released in the retina. The generation of lipid ROS is directly mediated by Fe^2+^ accumulation. In our study, MT conferred protection against ferroptosis by inhibiting the production of lipid ROS and reducing Fe^2+^ levels. Together with iron metabolism disorders, mitochondrial dysfunction is a major determinant of ferroptosis. Our study showed that the negative effects of RIR on mitochondrial morphology and function were attenuated by MT. According to these results, RIR led to increases in oxidative stress, intracellular iron accumulation, and lipid peroxidation in the retina, which resulted in RGC ferroptosis. Notably, MT supplementation ameliorated these changes and exerted pro–survival effects on neurons in RIR injury.

As RIR progresses, inflammation and the activation of retinal microglia profoundly contribute to pathological processes [48,49]. In addition, RIR injury has been demonstrated to be augmented and stimulated by macrophages [50,51]. In the present study, we found that the intravitreal administration of MT effectively suppressed microglial activation, macrophage infiltration, and proinflammatory cytokine levels after RIR. In addition, MT exerted a potent anti–apoptotic effect on RIR injury, as it decreased the number of TUNEL–positive cells in vivo and rescued the MMP in vitro.

Additionally, MT–induced signal transduction in the retina depends on MT receptor interactions. MT receptor levels have previously been verified in the retina after hypoxia–ischemia, and MT exerts its protective actions via both MT1 and MT2 [37]. Studies conducted in MT1 knockout (KO) mice showed that KO mice developed increased IOP and progressive RGC death [52]. Furthermore, another study indicated that MT binds to its membrane receptors to regulate the transcription factors p53 and p21 in response to oxidative stress [53]. We found that MT exerts a pro–survival effect and its antiferroptotic effect during RIR via both the MT1 and MT2 receptors. However, to determine how MT regulates the MT receptors in RIR, further investigation is needed and warranted.

Different transcription factors, including p53 [15], ATF3 [6], TAZ [54], and NFE2L1 [55], govern ferroptosis. The transcription factor p53 mediates neuronal death in a variety of stress–related and neurodegenerative conditions and is involved in cell survival and oxidative stress, thus regulating the progression of RIR injury [56,57]. In diabetic animals, system Xc–activity and GSH levels were found to be decreased in the retinas, whereas the oxidative stress level was increased [58]. Ma et al. [59] found that ubiquitin–specific peptidase 22 inhibited cardiomyocyte death induced by ferroptosis during myocardial IR injury via the SIRT1/p53/Slc7a11 axis. As previously studied, Alox12, an enzyme involved in the metabolism of fatty acids into hydroperoxides, is another key regulator of p53–dependent ferroptosis [36]. Studies have shown that Alox12 is indirectly activated by the inhibition of Slc7A11 transcription, resulting in ferroptosis via the accumulation of ROS [36]. In addition, it was previously observed that Alox12 is also critically involved in organ injury. One recent publication reported that Alox12 and its metabolite 12–hydroxyeicosatetraenoic acid led to the production of inflammatory mediators during hepatocyte ischemia [60]. Furthermore, a prior study noted that the underlying mechanism of CCl4–induced acute liver injury involved the activation of Alox12, which could be ameliorated using baicalein [61]. In this work, we elucidated the underlying mechanism through which MT affects and regulates p53–mediated ferroptosis, which has been the focus of recent studies on cell death.

## 5. Conclusions

Our present work revealed that MT suppresses RIR–induced ferroptosis by reducing the levels of lipid ROS and ferrous iron while increasing the levels of GSH via the regulation of the p53/Slc7a11/Alox12 signaling pathway. We provided convincing evidence showing that MT may be used as an effective drug for protection against RGC death. A possible limitation of this study is that these mechanisms were not explored in vitro due to the fragility and low yield of the primary RGCs after purification and culturing. In addition, MT exerted a preventive effect in our study, and an optimal dosing regimen of MT for RGC neuroprotection has not yet been established. It is unclear whether the delivery of MT can block retinal ferroptosis if administered after the RIR insult. Considering that the effects of MT could be influenced by the method of administration, further exploration of its therapeutic potential from a clinical perspective is warranted. Given its potential antiferroptotic properties, MT is a promising therapeutic agent for RGC protection. 

## Data Availability

All data generated or analyzed during this study are included in the article.

## Supplementary Materials

The following supporting information can be downloaded at: https://www.mdpi.com/article/10.3390/antiox12061173/s1, Figure S1: Changes of IOP during RIR.; Figure S2: Direct impact of MT on RGC survival.; Figure S3: MT inhibited RIR–induced oxidative stress via targeting p53; Table S1: Antibody list; Table S2: Sequences of the mouse primers; Table S3: Clinical characteristics of glaucoma donors.
