# Supplementary material for: Melatonin Alleviates Retinal Ischemia–Reperfusion Injury by Inhibiting p53–Mediated Ferroptosis
Source: Antioxidants (Basel). 2023 May 29;12(6):1173. doi: 10.3390/antiox12061173 (PMC10295547; doi:10.3390/antiox12061173)
Supplement: Supplementary file 1 [file antioxidants-12-01173-s001.zip › antioxidants-2262949-Supplementary materials.pdf]

Supplementary Information

Table S1. Antibody list.

Table S2. Sequences of the mouse primers.

Table S3. Clinical characteristics of glaucoma donors.

Figure S1. Changes of IOP during RIR.

Figure S2. Direct impact of MT on RGCs survival.

Figure S3. MT inhibited RIR-induced oxidative stress via targeting p53.

Table S1. Antibody list.

Antibody	Company	Catalog No.	Species	Specificity information	Application (dilution)
RBPMS	Abcam	ab152101	Rabbit	Mouse, Human	Immunofluorescence (1:200)
Tuj-1	BioLegend	801213	Mouse	Human, Mouse, Rat	Immunofluorescence (1:300)
Tuj-1	GeneTex	GTX130245	Rabbit	Human, Mouse, Rat	Immunofluorescence (1:300)
Brn3a	Santa Cruz	sc-8429	Mouse	Human, Mouse, Rat	Immunofluorescence (1:300)
Slc7a11	Abcam	ab175186	Rabbit	Human, Mouse, Rat	Western blotting (1:1000)<br>Immunoprecipitation (1:100)
Slc7a11	Novus	NB300-318SS	Rabbit	Human, Mouse, Rat	Immunofluorescence (1:100)
β-Actin	Cell Signaling Technology	#3700	Rabbit	Human, Mouse, Rat, Monkey, Dog	Western blotting (1:1000)
Fth1	Cell Signaling Technology	#3998	Rabbit	Human, Mouse, Rat, Monkey,	Western blotting (1:1000)
MDA	Abcam	ab243066	Mouse	Species independent	Western blotting (1:1000)
4-HNE	Abcam	ab48506	Mouse	Species independent	Immunofluorescence (1:50)

IL-1β	Santa Cruz	sc-52012	Mouse	Human, Mouse, Rat	Western blotting (1:1000)
Iba1	Cell Signaling Technology	#17198	Rabbit	Human, Mouse, Rat	Immunofluorescence (1:200)
F4/80	Abcam	ab100790	Rabbit	Human, Mouse	Immunohistochemistry (1: 1000)
p53	Cell Signaling Technology	#32532	Rabbit	Mouse, Rat	Western blotting（1: 1000）<br>Chromatin Immunoprecipitation (1:50)
Alox12	Santa Cruz	sc-365194	Mouse	Human, Mouse, Rat	Western blotting (1:1000)
Anti-mouse IgG	Cell Signaling Technology	#7076	House	Mouse	Western blotting （1: 3000）
Anti-Rabbit IgG	Cell Signaling Technology	#7074	Goat	Rabbit	Western blotting （1: 3000）
Alexa Fluor™ 488	Thermo Fisher Scientific	A-21206<br>A-21202	Donkey	Rabbit<br>mouse	Immunofluorescence (1:400)
Alexa Fluor™ 594	Thermo Fisher Scientific	A-21207<br>A-21203	Donkey	Rabbit<br>mouse	Immunofluorescence (1:400)
Alexa Fluor™ 647	Thermo Fisher Scientific	A-31573<br>A-31571	Donkey	Rabbit<br>mouse	Immunofluorescence (1:400)

Table S2. Sequences of the mouse primers.

	Forward	Reverse
Fth1	AACCGAGTCGTTCTGCCAAT	CTAGGGAGGGGACTGCTCAT
Ptgs2	CTGCGCCTTTTCAAGGATGG	GGGGATACACCTCTCCACCA
Slc7a11	GGTCAGAAAGCCAGTTGTGG	AGTATGCCCTTGGGGGAGAT
IL-1β	GGGCCTCAAAGGAAAGAATC	CTCTGCTTGTGAGGTGCTGA
IL-18	CTGTACAACCGCAGTAATACGG	ACTCCATCTTGTTGTGTCCTGG
β-Actin	GCCAACCGTGAAAAGAT	AGAGCATAGCCCTCGTAGAT
Slc7a11-site-1	CCTTACTTCCCCGGGTGTTT	GTCTGTTCATGCGGAGCTGT
GAPDH -site-1	AGTCCTATCCTGGGAACCATCACC	GCACGCACCAAGCGTGTG

Table S3 Clinical characteristics of glaucoma donors.

Eye	Sex	IOP (mmHg)	Condition	Visual acuity (BCVA)	Years with glaucoma diagnosis
R	female	40	Glaucoma (unknown)	no light perception	5
R	male	53	Glaucoma (CAG)	no light perception	10
L	male	55	Glaucoma (CAG)	no light perception	8

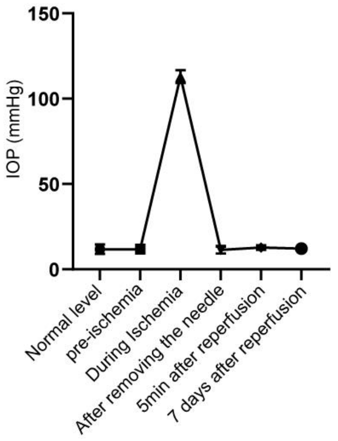

Figure S1 Changes of IOP during RIR injury.

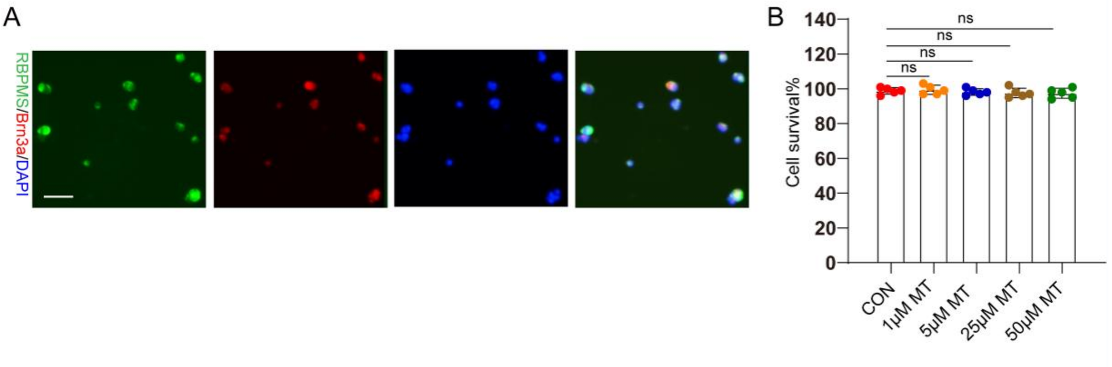

Figure S2. Direct impact of MT on RGCs survival. (A) Identification of mouse primary RGCs by double-immunofluorescence staining with RBPMS (green) and Brn3a (red) (scale bar = 25 μm). (B) CCK-8 assay was performed to test the effects of different concentrations of MT on the vitality of RGCs (n = 5). The colored dots on the graphs represent different types of interventions: CON (red), 1 μl MT (orange), 5 μl MT (blue), 25 μl MT(brown), and 50 μl MT (green). All the results are presented as the mean ± SD and were analyzed by one-way ANOVA followed by Tukey's post hoc test. ns = not significant.

.

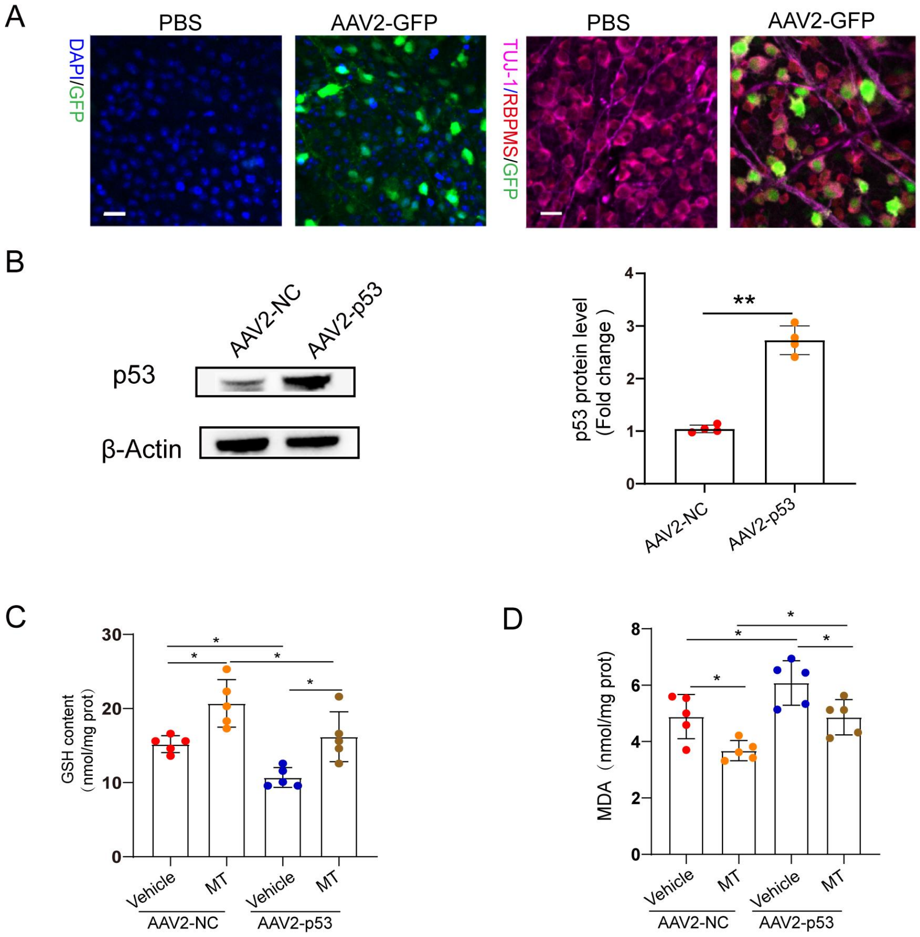

Figure S3 MT inhibited RIR-induced oxidative stress via targeting p53. (A) GFP expression (green) was identified in the retinal flat mounts. The transfection efficacy in RGCs was confirmed by colabeling with the RGC-specific markers Tuj-1 (pink) and Brn3a (red) after AAV2-p53 injection (scale bar = 50 μm). (B) Western blotting analysis of AAV2-mediated p53 expression (n = 4). Data are shown as mean ± SD and were analyzed by Two-tailed Student's t test. **$p < 0.01$. (C-D) Oxidative stress indicator levels, including GSH and MDA (n= 5). The colored dots on the graphs represent data points for different individuals: AAV2-NC (red), and AAV2-p53 (orange). Data are shown as mean ± SD and were analyzed by one-way ANOVA followed by Tukey's post hoc test. *$p < 0.05$, **$p < 0.01$
